# Heat-Related Illness Is Associated with Lack of Air Conditioning and Pre-Existing Health Problems in Detroit, Michigan, USA: A Community-Based Participatory Co-Analysis of Survey Data

**DOI:** 10.3390/ijerph17165704

**Published:** 2020-08-07

**Authors:** Jacqueline E. Cardoza, Carina J. Gronlund, Justin Schott, Todd Ziegler, Brian Stone, Marie S. O’Neill

**Affiliations:** 1 School of Public Health, University of Michigan, Ann Arbor, MI 48109, USA; jcardoza@umich.edu (J.E.C.); marieo@umich.edu (M.S.O.); 2 Survey Research Center, Institute for Social Research, University of Michigan, Ann Arbor, MI 48104, USA; 3 EcoWorks, Detroit, MI 48219, USA; jschott@ecoworksdetroit.org; 4 Environmental Health Sciences, University of Michigan School of Public Health, Ann Arbor, MI 48109, USA; toddz@umich.edu; 5 Georgia Institute of Technology School of City and Regional Planning, Atlanta, GA 30332, USA; stone@gatech.edu

**Keywords:** climate change, heat wave, heat exhaustion, community-based participatory research

## Abstract

The objective of the study was to investigate, using academic-community epidemiologic co-analysis, the odds of reported heat-related illness for people with (1) central air conditioning (AC) or window unit AC versus no AC, and (2) fair/poor vs. good/excellent reported health. From 2016 to 2017, 101 Detroit residents were surveyed once regarding extreme heat, housing and neighborhood features, and heat-related illness in the prior 5 years. Academic partners selected initial confounders and, after instruction on directed acyclic graphs, community partners proposed alternate directed acyclic graphs with additional confounders. Heat-related illness was regressed on AC type or health and co-selected confounders. The study found that heat-related illness was associated with no-AC (*n* = 96, odds ratio (OR) = 4.66, 95% confidence interval (CI) = 1.22, 17.72); living ≤5 years in present home (*n* = 57, OR = 10.39, 95% CI = 1.13, 95.88); and fair/poor vs. good/excellent health (*n* = 97, OR = 3.15, 95% CI = 1.33, 7.48). Co-analysis suggested multiple built-environment confounders. We conclude that Detroit residents with poorer health and no AC are at greater risk during extreme heat. Academic-community co-analysis using directed acyclic graphs enhances research on community-specific social and health vulnerabilities by identifying key confounders and future research directions for rigorous and impactful research.

## 1. Introduction

Global climate change is occurring, attributable in large part to the increased burning of fossil fuels by humans [1]. Extreme heat events are expected to increase in frequency, severity, and duration as a result of climate change [1]. Warm temperature extremes have direct impact on health by compromising the body’s ability to regulate its internal temperature [2]. Loss of internal temperature control can result in a cascade of health effects including heat cramps, heat exhaustion, heatstroke, hyperthermia, respiratory problems, or mortality [2]. The impacts of extreme heat events disproportionately affect the health of certain subpopulations. Vulnerable populations such as the elderly, young children, low-income communities, communities of color, and individuals with existing health problems have a higher health burden of heat exposure [3,4].

The location of populations can also further heighten the burden of heat-related illness and mortality. Populations who live in urban areas are at a disadvantage given the urban infrastructure. Three urban features can contribute to what is known as the urban heat island effect, in which urban and suburban areas have elevated air temperatures relative to surrounding rural areas or wildlands. These include use of dark, dense paving and building materials; the three-dimensional form of buildings, which absorb solar radiation and restrict air circulation; and a reduced abundance of vegetation, which decreases shade and restricts evaporation and transpiration, which can cool the environment [5]. On hot days, which tend to occur with high-pressure weather systems, air quality in urban areas may also worsen [2]. In areas where air pollution exposures tend to be higher, adverse health effects on extreme heat days could potentially be worse than they are in areas with lower levels of air pollution [3]. People living in urban areas may find refuge from extreme heat through the use of cooling mechanisms such as air conditioning (AC). However, AC units can increase the outdoor air temperature. For example, in Tokyo the “waste” heat from AC units has caused a temperature rise of 1–2 °C or more on weekdays [6]. The increase of outdoor heat can promote further increased use of AC for some. However, lower-income residents typically lack the monetary resources to obtain AC or may limit using AC to minimize utility costs [5].

This study uses data from Detroit, Michigan, a U.S. city that has a 35.7% poverty rate [7] and a median household income of $28,099 [7], where health disparities related to heat exposure are well documented. The data were collected in Detroit as part of a National Science Foundation (NSF)-funded three-site project “Enhancing Emergency Preparedness for Critical Infrastructure Failure during Extreme Heat Events.” Previous studies [8,9] showed that Detroiters experience high indoor temperatures, use relatively less AC compared to other U.S. cities, lack access to cooling centers and cool spaces, and experience pronounced urban heat island effects. In interviews, Detroit residents described their concern about heat and other climate-related exposures [10,11].

When researching environmental and social determinants of marginalized communities’ health status, researchers and practitioners have called for increased attention to greater community involvement and sensitivity about working in diverse communities [12]. Recently, community-based approaches to public health are becoming a more commonly employed approach to develop knowledge and action in the field [13]. Community-based participatory research (CBPR) focuses on conducting research with a community as a social and cultural entity that is actively engaged in the research process. Partnerships form to strengthen and enhance the understanding of the research interests through utilizing the unique skills, resources, and knowledge of everyone involved. This collaborative approach treats all partners as equals and integrates knowledge and action for the mutual benefit of all partners.

With the rise of CBPR within the public health field, researchers are now challenged to incorporate community members as equal partners in all phases of research. However, community partners often do not participate in the analysis phase of the research [14] despite the potential for alternate perspectives on data interpretation to enhance research insights [15,16]. An analysis of 25 National Institutes of Health (NIH)- and Centers for Disease Control and Prevention (CDC)-funded CBPR projects in the Southeast U.S. found a wide range of community participation rates among the components of research, with data analysis, data ownership, and interpretation of findings ranked among the lowest in terms of community participation [17]. Additionally, sharing research results with community partners through internal dissemination requires that complex data be discussed in an understandable format with opportunities to answer questions, exchange knowledge, jointly interpret results, and discuss how the results will be used [18]. 

For this study, we employed a CBPR approach to co-analyze and co-interpret the data from a larger project on self-reported experiences with housing and community resources and health impacts of extreme heat among 101 residents of Detroit, Michigan. The research objectives were to (1) understand the association between heat-related health effects and AC ownership, (2) understand the association between heat-related health effects and health status, and (3) gain new insights on the inclusion of community partners in the data analysis and interpretation phase of research.

## 2. Methods

### 2.1. Survey Data Collection

We obtained consent from and surveyed 101 adult Detroit residents in 2016–2017 regarding weather and air pollution. Fifty-one of these residents were selected from lists of individuals who had received services from or volunteered with three neighborhood organizations--Jefferson East Inc. (multi-service neighborhood organization that serves low-income populations on Detroit’s east side), the Friends of Parkside (resident advocate based at the Villages of Parkside public housing complex on Detroit’s east side), and Southwest Detroit Environmental Vision (neighborhood organization focused on improving the environment and strengthening the economy of Southwest Detroit). List members were categorized by housing type (masonry vs. non-masonry, single vs. multi-family, two or fewer stories vs. 2–5 stories, presumed AC status) and contacted randomly within type. An additional 50 Detroit residents were recruited without consideration of neighborhood or housing type at community events. The selected residents were asked 125 questions about their demographics, housing address and characteristics, weather and air pollution risk perception, electrical cooling behaviors, short-term cooling strategies in and outside the home, long-term cooling strategies including home improvements, black-out experiences, social networks, general health status, and heat impacts on health. The survey was administered, after the resident’s informed consent, by study staff on a tablet with programmed skip patterns. All subjects gave their informed consent for inclusion before they participated in the study. The study was conducted in accordance with the Declaration of Helsinki, and the protocol was approved by the University of Michigan Institutional Review Board (HUM00102979).

### 2.2. Health Outcome

The health outcome of interest, heat-related illness, or “heat exhaustion”, was measured through the survey question, “During the past 5 years or so, have you had medical symptoms related to heat exhaustion from high temperatures such as muscle cramps, dizziness, tiredness, weakness, throbbing headache, nausea or vomiting, fainting, or paleness?” using a definition adopted from the CDC [19].

### 2.3. Main Exposures 

AC use was measured through the survey question, “Do you use the air conditioner to cool your home during the summer?”. We subcategorized responses as: those who did not use or do not have central air or a window AC unit (none), those who program their thermostats for their central air (reference), and those who have a window AC unit (low).

Health status was measured through the survey question, “In general, compared to other people your age, would you say your health is…” where the designated options were Excellent, Good, Fair, or Poor.

### 2.4. Co-Analysis 

**Figure 1:**
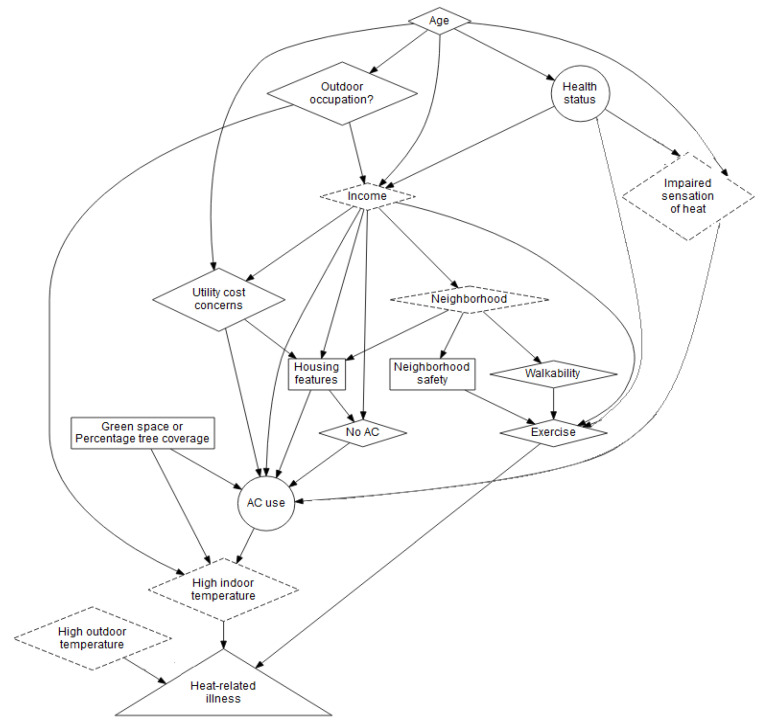
Directed acyclic graph (DAG) of proposed associations between and confounding of air conditioning (AC) use and heat exhaustion and self-reported health status and heat exhaustion.

A directed acyclic graph (DAG) was used to illustrate the relationships between the variables and to identify potential confounders and covariates. DAGs are a graphical tool used in epidemiology to identify, a priori, sources of bias and hence variables that should and should not be included in a model of the association between a particular health outcome and a particular exposure. Arrows represent direct effects, and the graph is “acyclic” because there are no feedback loops in the graph. In the final model, controlling for or conditioning on a variable closes paths that pass through that variable and, in theory, eliminates bias from that variable, with the goal of having only causal paths (which may still include mediators) from the exposure to the outcome. (For further discussion including instances when bias can actually be introduced by conditioning on a variable, such as a collider, see Greenland and Pearl’s 2014 overview of DAGs [20].) Four academic partners first constructed a DAG and then provided instruction to the community partners on the concept and function of DAGs. Next, the DAG was brought to a meeting of the “Heatwaves, Housing, and Health” community–academic research partnership. This partnership’s steering committee included University of Michigan faculty and representatives from five not-for-profit organizations in Detroit, described in further detail [21]. Three of these organizations, Jefferson East, Inc., Friends of Parkside, and Southwest Detroit Environmental Vision, assisted with recruitment and retention (see above). The other two organizations, Detroiters Working for Environmental Justice and EcoWorks, advocate for and assist with environmental and housing concerns on behalf of Detroit residents citywide. Through discussion, the community partners and academic partners co-selected potential confounders, expanding the original DAG the academic partners created to include housing and neighborhood characteristics, utility cost concerns, and age (Figure 1). The community partners emphasized the potential for negative confounding from the fact that older homes with more stories and large homes that have been converted to multifamily housing may not have central AC (correlation with the exposure) but may have less need for it given the cooling impacts of the design features of these older homes and resulting potential association with indoor temperature and, therefore, the health outcome.

Variables in circles were the main exposure, the variable in the triangle was the health outcome, variables in diamonds were potential confounders, variables in boxes were potential confounders selected by community partners, variables in dashed diamonds were variables for which information was not available or, in the case of neighborhood, indicators that we could not include in the models because some neighborhoods were represented by only a single respondent.

### 2.5. Potential Confounders 

Utility cost concerns were measured through the survey question, “When it comes to air conditioning, the cost of electricity is…?” where the designated options were Very, Somewhat, Not too, and Not at all limiting. We included this variable due to concerns that individuals with central or window AC would not fully use these features if utility cost concerns were high.

Indoor environments may remain cool in this climate for housing-related reasons besides AC ownership, such as proximity of the roof to living spaces or surrounding shade. Therefore, housing stories, housing family size, and the percent of tree canopy were included as potential confounders. Tree canopy from the 2010 Southeast Michigan Council of Governments (SEMCOG) high-resolution land cover data created by Light Detection and Ranging (LIDAR) was summarized by 2010 census tract and City of Detroit parcel boundaries using the Tabulate Intersection tool in ArcGIS 10.6.

Given that individuals may be more likely to have heat illness due to outdoor exposure if their neighborhood is walkable, we included measures of physical walkability and neighborhood safety. Walk Score measures the walkability by awarding points based on the distance from each respondent’s address to a category of amenities [22]. Amenities within a 5 min walk (0.25 miles) are awarded the most points and no points are given if they are more distant than a 30 min walk. The Walk Score compiles data from sources including Google, Education.com, Open Street Map, the U.S. Census, Localeze, and places added by the Walk Score user community. Neighborhood safety was measured through the survey question, “How safe or unsafe do you feel in your neighborhood?”.

### 2.6. Statistical Analysis 

Coding and analysis of the variables was conducted in SAS version 9.3 and R version 3.4.3. Linearity was confirmed visually through plots of natural cubic splines of each variable, and bivariate correlations and variance inflation factors (VIFs) were assessed. The web tool DAGitty [23] was used to visualize the causal relationships between the heat exhaustion outcome and the two main exposures, AC use and health status, and identify which variables should and should not have been included in each model to adequately control for confounding of the exposure–health outcome association. Multicollinearity between covariates was assessed using VIFs.

## 3. Results

Forty-eight residents reported experiencing heat exhaustion, 52 residents reported not experiencing heat exhaustion in the last 5 years, and one resident did not know. Among those who reported experiencing heat exhaustion, 17 (35%) did not have any AC, 24 (50%) had window AC units, and 7 (15%) had central AC (Table 1). The respondents who reported having no AC had 3.78 times the odds of experiencing heat exhaustion compared to respondents who had central AC (*p* = 0.03) (Table 1). A similar increase in the odds of experiencing heat exhaustion was seen for those having no AC compared to those who had central AC among respondents who lived in their homes for 5 years (odds ratio (OR) = 9.16, *p* = 0.01). Additionally, the respondents who reported having poor/fair health status had 2.88 times the odds of experiencing heat exhaustion compared to respondents who had excellent/good health status (*p* = 0.01) (Table 1). Respondents who felt their neighborhood was very/somewhat unsafe had 4.24 times the odds of experiencing heat exhaustion compared to respondents that felt their neighborhood was very/somewhat safe (*p* = 0.04). All VIFs were under 2, indicating an absence of multicollinearity. The mean age for those living in their home for at least 5 years was 51.52 years old (Table 2).

To block non-causal pathways in the DAG (Figure 1) between heat exhaustion and AC, age, health status, percent tree canopy, house stories, family size, and utility cost concerns needed to be included in the regression model. After doing so (model 1, Table 3), the odds of heat exhaustion among those with window AC (1.43, 95% confidence interval (CI) = 0.42, 4.82) and with no AC (3.35, 95% CI = 0.80, 14.08) were not significantly higher than the odds of heat exhaustion compared to those with central AC. 

In Model 2, in which we controlled for the percent of trees in the parcel instead of in the tract, the main exposure, AC use, was not statistically significant for either level compared to respondents with central AC. The odds of experiencing heat exhaustion among those with window AC were 1.41 (95% CI = 0.42, 4.79) and with no AC they were 3.27 (0.76, 14.00) compared to those with central AC.

Given that heat exhaustion was asked in a 5 year timeframe, Model 3 was restricted to individuals residing in the same home for the previous 5 years. Model 3 shows that window AC use was not significantly associated with heat exhaustion, but no AC use was associated with heat exhaustion. However, the confidence intervals were very wide.

For Model 4, from examination of the DAG, only one variable, age, needed to be included in the model because control for this variable blocked the paths between potential confounders of the association between the main exposure, health status, and heat exhaustion. (Note that many of the other variables in the DAG are mediators, or are on the causal pathway, between health status and heat exhaustion and should therefore not be controlled for when estimating the total, or direct + indirect, effect.) In the final model, self-reported health status was statistically significantly associated with heat exhaustion. The odds of experiencing heat exhaustion among people with fair/poor health vs. those with good/excellent health was 3.15 (95% CI = 1.33, 7.48). 

In Model 5, the association between heat exhaustion and AC use was analyzed while adjusting for only health status and age. The odds of experiencing heat exhaustion among those with window AC were 2.02 (95% CI = 0.64, 6.39) and with no AC they were 4.55 (1.22, 17.72) compared to those having central AC.

Model 6 is similar to Model 5 but restricted to individuals residing in the same home for the previous 5 years. The odds of experiencing heat exhaustion among those with window AC were 3.84 (95% CI = 0.65, 22.83) and with no AC they were 12.47 (1.63, 95.21) compared to central AC.

## 4. Discussion

Among Detroit residents, not having central AC was associated with increased odds of heat exhaustion, as hypothesized, when the dataset was restricted to the 57 people who lived in their homes for at least 5 years. However, the wide confidence intervals suggested that the sample size was insufficient to examine this relationship with adequate adjustment for relevant confounders. In contrast, the DAG exercise did not identify a need to control for housing characteristics in assessing the association between health status and heat exhaustion. Therefore, our finding of a 3.15-times increased odds of heat exhaustion among individuals with fair/poor health compared to those with good/excellent health, without reason to exclude individuals who had changed residences in the previous 5 years from the analysis, was more robust. Our findings regarding AC and heat-related illness were consistent with findings in a recent survey of New York City, New York, USA residents. In this survey, low-income individuals had a 3.1-times higher odds (95% CI: 1.8, 5.5) of not having AC, and in turn, these low-income individuals were more likely to be concerned that heat could make them ill and that climate change would affect their health than participants with a higher household income, OR = 1.6 (95% CI: 1.0, 2.3) [24]. 

We did not examine associations between the number of individuals living in the home, utility cost concerns, and neighborhood safety, respectively, and heat exhaustion in controlled analyses. However, their associations with heat exhaustion in unadjusted analyses suggested that our decision to control for these characteristics was justified. Furthermore, these associations would likely benefit from analysis with a larger sample size, and we look forward to analyzing them with the other cities, Phoenix and Atlanta, in the study.

In addition to contributing to the identification of relevant new variables for the analysis, community partners highlighted the practical difficulties in using the CDC-based definition to define the heat exhaustion outcome. The community partners questioned whether residents understood the health-based terms in the definition well enough to personally identify with the experience named “heat exhaustion” and appropriately respond to the question. For example, heat discomfort and exhaustion experienced with high frequency was potentially not perceived by some of our respondents as a medical problem. Alternatively, the fairly broad definition provided may have resulted in over-reporting of heat exhaustion. Community partners suggested that future research should use more relatable terms such as “feeling too hot” instead of technical terms. The exposure, AC use, also may not have been communicated in terms that community members would understand, as the community partners indicated that some residents may not have been aware of whether their housing had central AC.

The DAG co-construction exercise, a CBPR co-analysis technique, proved highly useful, and we recommend this approach to enhance the analysis phase of research, from which community partners are often excluded. Although it took additional time and energy for the community and academic partners to meet and discuss the DAG, this approach highlighted the unique and critical perspective community members have in regard to environmental exposures and health outcomes as well as the more general benefits of taking the time to discuss a complex analysis with a team possessing a broad range of expertise and local knowledge. The goals of the analysis aligned with the concerns of the community partners as the health outcome was a non-emergency outcome, not morbidity or emergency department visits as is often researched in secondary analyses. An outdoor environmental exposure, neighborhood safety, was included as a confounder as a result of the input from community partners. Local knowledge of the benefits of certain housing features in keeping indoor environments cooler resulted in inclusion of potential negative confounders—larger multi-family homes with more stories—that, by virtue of their age and the incomes of the residents, may not have central AC but may still remain cool on hot days.

In the months between the survey design, data collection, and analysis, community partners expressed increased interest in underlying health causes of heat exhaustion (relating to the results of Model 4) and the uncertainty remaining around the strength of association between AC use and heat exhaustion (Model 1) given the sample size. These questions from the community members reflect the shared interest and pursuit of better understanding exposures and their associations with health outcomes and suggest topics of future research. CBPR co-analysis can enhance epidemiologic analyses of community-specific social and health vulnerabilities to reach enhanced understanding.

## 5. Conclusions

Pre-existing self-reported health status was associated with heat-related illness. Access to central AC was also associated with heat-related illness, so consideration of this factor for interventions is warranted, especially given the consistency of this finding with other literature. The community partners, as experts in climate, housing, and health issues in Detroit, provided important additional confounders during the co-construction of a DAG, indicating that DAG co-construction is a useful CBPR co-analysis technique. Additional topics and future research questions discussed with the community partners focused on social determinants of health and environmental conditions that cause health concerns or exacerbate existing health conditions.

## Figures and Tables

**Table 1:** Counts, means, and relative risks of experiencing vs. not experiencing heat exhaustion in the previous five years by demographic, housing, and neighborhood characteristics for all study participants who knew whether they had experienced heat exhaustion in the previous 5 years (*n* = 100) and for those residing in their home in the previous 5 years (*n* = 61) in Detroit, MI, 2016–2017.

Variable	Heat Exhaustion		No Heat Exhaustion		Relative Risk (95% CI)	*p*-Value ^a^
	*n*	%	*n*	%		
Total	48	47	52	53		
Total—in home for 5 years	26	43	35	57		
AC use						
No	17	35	10	19	3.78 (1.13, 13.13)	0.03
Window	24	50	26	50	2.09 (0.73, 6.39)	0.21
Central (reference)	7	15	16	31	1.00	NA
AC use—in home for 5 years						
No	10	38	6	17	9.16 (1.40, 75.35)	0.01
Window	14	54	17	49	4.78 (0.96, 34.47)	0.09
Central (reference)	2	8	12	34	1.00	NA
Health Status						
Poor/Fair	26	54	15	29	2.88 (1.24, 6.80)	0.01
Good/Excellent (reference)	22	46	37	71	1.00	NA
House Stories						
1 (reference)	5	10	2	4	1.00	NA
1.5	18	37	13	2	0.56 (0.07, 3.53)	0.68
2–3	21	44	36	69	0.24 (0.03, 1.30)	0.11
4	4	8	1	2	1.54 (0.09, 57.66)	1.00
House Stories—in home for 5 years						
1 (reference)	3	11	2	6	1.00	NA
1.5	9	35	7	20	0.86 (0.09, 7.14)	1.00
2–3	14	54	26	74	0.37 (0.41, 2.63)	0.35
4	0	0	0	0	NA	NA
Housing family number						
Multiple	10	21	20	38	0.42 (0.17, 1.06)	0.08
Single (reference)	38	79	32	61	1.00	NA
Utility Costs Concerns						
Somewhat/very limiting	21	44	33	63	0.45 (0.19, 1.01)	0.07
Not too/not at all limiting	27	56	19	36	1.00	NA
Neighborhood Safety						
Somewhat/Very unsafe	10	21	3	6	4.24 (1.09, 19.14)	0.04
Somewhat/Very safe (reference)	38	79	49	94	1.00	NA
Neighborhood Safety—in home for 5 years						
Somewhat/Very unsafe	4	15	3	9	1.92 (0.38, 10.64)	0.45
Somewhat/Very safe (reference)	22	85	32	91	1.00	NA

NA = not applicable. **^a^** Fisher’s exact test. CI: confidence interval.

**Table 2:** Means and t-tests of experiencing vs. not experiencing heat exhaustion in the previous five years by respondent age and neighborhood characteristics for all study participants providing addresses (*n* = 101) and for those residing in their homes in the previous 5 years (*n* = 61) in Detroit, MI, 2016–2017.

Variable	*n*	Min.	Max.	Heat Exhaustion		No Heat Exhaustion		Difference (95% CI) ^a^	*p*-Value ^a^
				Mean	SD	Mean	SD		
Age	97	18	77	44.30	0.90	49.96	0.90	1.83 (−0.50, 11.83)	0.07
Percent Tract Trees	101	2.40	31.08	14.63	0.81	14.86	2.08	0.22 (−1.86, 2.33)	0.83
Percent Parcel Tree	101	0	74.07	17.04	2.48	12.88	2.54	−1.04 (−12.11, 3.80)	0.30
Walk Score	101	23	78	57.73	1.20	54.81	2.41	−1.32 (−7.31, 1.47)	0.19
Age–in home for 5 years	57	19	77	51.52	0.83	54.75	0.77	0.89 (−4.12, 10.58)	0.38
Percent Tract Trees–in home for 5 years	61	3.66	25.66	14.99	0.57	14.83	1.83	−0.11 (−3.00, 2.68)	0.91
Percent Parcel Tree–in home for 5 years	61	0	74.07	19.59	2.17	13.92	0.99	−1.05 (−16.50, 5.17)	0.30
Walk Score–in home for 5 years	61	23	78	59.11	0.81	53.54	2.01	−1.78 (−11.87, 0.73)	0.08

**^a^** Independent two-group t-test.

**Table 3:** Associations (odds ratios and 95% confidence intervals) of heat exhaustion with main exposures AC use and poor/fair health status for the study participants in Detroit, MI, 2016–2017.

Model Number	*n*	Window AC	No AC	Health Status (Poor/Fair)
Model 1 ^a^	96	1.43 (0.42, 4.82)	3.35 (0.80, 14.08)	--
Model 2 ^b^	96	1.41 (0.42, 4.79)	3.27 (0.76, 14.00)	--
Model 3 ^c^	57	3.38 (0.50, 22.58)	10.39 (1.13, 95.88)	--
Model 4 ^d^	97	--	--	3.15(1.33, 7.48)
Model 5 ^e^	96	2.02 (0.64, 6.39)	4.66 (1.22, 17.72)	--
Model 6 ^f^	57	3.84 (0.65, 22.83)	12.47 (1.63, 95.21)	--

^a^ Heat Exhaustion = Window AC + No AC + Age + Health Status + Percent Tract Trees + House Stories + Family Size + Utility Costs. ^b^ Heat Exhaustion = Window AC + No AC + Age + Health Status + Percent Parcel Trees + House Stories + Family Size + Utility Costs. ^c^ Same model as model 2 but restricted to people who lived in their home for at least 5 years. ^d^ Heat Exhaustion = Health Status (Poor/Fair) + Age. ^e^ Heat Exhaustion = Window AC + No AC + Age + Health Status. ^f^ Same model as model 5 but restricted to people who lived in their home for at least 5 years.
